# From transmission to adaptive evolution: genomic surveillance of Getah virus

**DOI:** 10.3389/fcimb.2025.1513392

**Published:** 2025-06-04

**Authors:** Yuge Yuan, Yujia Hao, Chengcheng Peng, Duo Zhang, Wenzhou Ma, Pengpeng Xiao, Nan Li

**Affiliations:** ^1^ Wenzhou Key Laboratory for Virology and Immunology, Institute of Virology, Wenzhou University, Wenzhou, China; ^2^ College of Veterinary Medicine, Jilin University, Changchun, China

**Keywords:** Getah virus, epidemiological situation, host range, cross-species, adaptive evolution

## Abstract

Getah virus (GETV) is a member of the *Alphavirus* of the *Togaviridae*. It is a single-stranded positive-RNA virus that is mainly transmitted by mosquitoes. In recent years, the spread of GETV has become increasingly serious, causing serious losses to the animal economy and posing a potential threat to public health. GETV infected animals extend from traditional domestic animals such as horses and pigs to cattle, foxes and other animals. Especially in China, the virus has been detected in many provinces in recent years. In addition, GETV-specific antibodies were detected in healthy humans. However, the threat posed by GETV in China has not received enough attention. In this study, we downloaded all available GETV genome-wide serials (82 serials in total) from the NCBI as of December 2023. We integrate multiple bioinformatics approaches to understand the characteristics of GETV from the perspectives of epidemiology, virus-host co-evolution, and viral adaptation analysis. The results of this study show that GETV is rapidly expanding its host range and geographical distribution at high evolutionary rates due to the lack of commercially available vaccines. Second, we clearly reveal the cross-species transmission of GETV. Finally, we identified important adaptive and active selection sites. GETV and its media are widely distributed in China, and new host infections continue to appear. Therefore, strengthening surveillance and prevention to avoid serious losses to the pandemic is an important task we face today.

## 1 Introduction

Getah virus (GETV) is a member of the *Alphavirus* within the *Togaviridae* (Zhang et al., 2020). In 1955, GETV was first isolated from *Aedes albopictus* living in rubber plantations in Malaysia, and it subsequently spread widely across island countries in the South Pacific region. Since its isolation, GETV has been found in various vertebrates, including humans, monkeys, pigs, horses, and other mammals. Infections in these species are crucial for maintaining the transmission cycle of zoonotic diseases like GETV in nature (Scherer et al., 1962). Studies have shown (Li et al., 2022) that GETV can be amplified by mosquito-vertebrate host-mosquito circulation and infected horses, pigs, and cattle. GETV infects pigs of all ages, especially newborn piglets, resulting in high morbidity, mortality and severe economic losses.

GETV is an enveloped, single-stranded positive-sense RNA virus primarily transmitted by mosquitoes. The virus’s genome is 11–12 kb in length, containing untranslated regions (5’ UTR and 3’ UTR) at both ends. The 5’ end features a methylated cap structure (7-methylguanosine), and the 3’ end has a polyadenylated tail. The genome consists of two open reading frames (ORFs). The first ORF occupies about two-thirds of the genome and encodes four non-structural polyproteins (NSP1 to NSP4), which are responsible for RNA transcription, replication, polyprotein cleavage, and RNA capping. This region is followed by the 26S RNA junction region, which facilitates the transcription of subgenomic 26S RNA within the cell. The second ORF, occupying about one-third of the genome, encodes the structural proteins Cap, E3, E2, 6K, and E1. The capsid protein comprises two distinct structural domains, the N-terminal and the C-terminal. The C-terminal domain is highly conserved across all *Alphavirus* capsid proteins, while the N-terminal domain is highly variable. E2 is the primary protein mediating the virus’s entry into host cells during infection (Chen et al., 2018; Carey et al., 2019; Wang et al., 2022).

The seroepidemiology of GETV-infected animals has attracted attention due to the outbreak of GETV in animals such as horses and pigs, among which the most detailed investigation and study has been conducted in Japan, and in 1972, a serological study was conducted in local horses in Japan, and serum neutralization tests showed that the positive rates of GETV antibodies in local horses before and after the outbreak were 6% (14/232) and 61.2% (172/282), respectively (Imagawa et al., 1981). Serological surveys indicate (Zhang et al., 2020) that GETV-specific antibodies have been detected in the sera of humans, pigs, horses, cattle, goats, dogs, rabbits, kangaroos, chickens, and wild birds from countries in Asia, Europe, and Oceania. For a long time, GETV was considered pathogenic only to pigs and horses, causing fever, widespread rashes, hindlimb edema in horses, arthritis in piglets, and reproductive disorders in sows (Fukunaga et al., 2000; Bannai et al., 2019). Several outbreaks of diseases caused by GETV infections in pigs and horses have been reported in Japan and India (Nemoto et al., 2015). In China, the first GETV strain was isolated from *Culex gelidus* mosquitoes in Hainan Province in 1964 and was named M1 (Zhai et al., 2008). In our study, we illustrate the cross-species transmission of GETV that has been taking place in recent years through a visualization of the relationship between GETV whole genome sequences and hosts in the NCBI database.

Viruses are a potential threat to global epidemics and future pathogens (Thakur et al., 2024). GETV is a neglected zoonotic amorphous virus. From a surveillance perspective, the silent circulation of GETV in asymptomatic livestock and wild animal hosts makes the early warning system more complex. In addition, the threat posed by the expanding host range and geographical transmission of the GETV need to be paid attention. Since the early 21st century, the geographic distribution of GETV has expanded, with new transmission vectors emerging, an increasing number of infected animals, and more frequent outbreaks. This has resulted in significant economic losses and poses a potential threat to public health (Li et al., 2022). GETV-specific antibodies were detected in healthy people and patients with fever of unknown cause, suggesting that the scope of GETV infection was expanding, and the impact on public health could not be ignored. Due to the public health importance of GETV (Zhang et al., 2020), this study investigates the epidemiology, host diversity, and adaptive evolution of GETV based on whole genome sequences downloaded from NCBI.

## 2 Materials and methods

### 2.1 Sequence selection

We retrieved complete GETV genome sequences from the NCBI GenBank database (https://www.ncbi.nlm.nih.gov/) up to December 2023. Relevant information such as GenBank accession number, host, country, region, and collection date (year) was extracted. Based on this information, we analyzed the geographic distribution of GETV in Asia, and the relationship with hosts, and conducted analyses on the transmission, mutation, and evolution of GETV.

### 2.2 Spatial Distribution of GETV

The downloaded data from NCBI was summarized based on GenBank accession number, host, country, region, and collection date (year) (**Supplementary Data**). Using this data, a map of GETV distribution in Asia was created. The Asian distribution map was drawn using the online tool MapChart (https://www.mapchart.net/), which allows the selection of different map types and region coloring and supports downloading the final map image.

### 2.3 Distribution of susceptible hosts of GETV

Based on the sequence information downloaded from NCBI, sequences were classified by country, province, and host type. Cartoon images of different hosts were downloaded from the Biorender website (https://www.biorender.com/) and used to create a distribution map of susceptible hosts using Microsoft Office PowerPoint, based on the sorted sequence information.

### 2.4 Cross-species analysis of GETV and hosts

To evaluate the co-phylogenetic relationships between GETV and its hosts, we compared the phylogenetic trees of the virus and its hosts. Firstly, we constructed a phylogenetic tree using 82 GETV whole genome sequences from the NCBI database. Multiple sequence alignment (MSA) was performed using MAFFT v7.543 (Katoh et al., 2002), visually inspected, and manually edited using Aliview v1.27 (Larsson, 2014). After obtaining the MSA, the phylogenetic tree of the virus was constructed using IQ-TREE v1.6.12 (Nguyen et al., 2015) with 1000 bootstrap replicates. Based on the BIC value calculated using MEGA11, the best-fit nucleotide substitution model was GTR+G+I. Visualization and annotation of the tree were done using Figtree v1.4.4 (http://tree.bio.ed.ac.uk/software/figtree/). Coloring of the phylogenetic tree was done using the iTOL online tool (Letunic and Bork, 2021) (https://itol.embl.de/). For constructing the host phylogenetic tree, mitochondrial cytb sequences of hosts were downloaded from the NCBI database and processed similarly using MAFFT v7.543 (Katoh et al., 2002), manually edited in Aliview v1.27 (Larsson, 2014), and a phylogenetic tree was constructed using IQ-TREE v1.6.12 (Nguyen et al., 2015) with the best-fit BIC model TPM2u+F+R3. The co-phylogenetic relationships between the virus and host trees were manually annotated in Microsoft Office PowerPoint.

### 2.5 Transmission and adaptive evolution of GETV

To illustrate the routes of horizontal and vertical transmission of GETV, a transmission route map was created using the Biorender online tool (https://www.biorender.com/). For analyzing amino acid variations in GETV, BioAider v1.527 (Zhou et al., 2020) was used to analyze amino acid mutation sites across the whole genome sequences. The first original strain MM2021 uploaded in 1955 was used as a reference for amino acid mutation analysis. MSA was performed using MAFFT v7.543 (Katoh et al., 2002), and the aligned nucleotide sequences were translated into amino acid sequences in Aliview v1.27 (Larsson, 2014). The amino acid sequences were then analyzed for mutation sites and visualized as lollipop plots using BioAider v1.527 (Zhou et al., 2020). To further identify positively selected sites, the whole genome sequences of GETV were analyzed using the datamonkey online tool (http://www.datamonkey.org/) for positive selection site prediction. Selection pressure at sites was detected using several methods: MEME (Mixed Effects Model of Evolution), FEL (Fixed Effects Likelihood), FUBAR (Fast, Unconstrained Bayesian Approximation), and SLAC (Single-Likelihood Ancestor Counting), MEME uses a mixed-effect maximum likelihood method to test the hypothesis that a single locus is affected by contextual positive selection; FEL assumes that the selection pressure at each locus is constant throughout phylogenesis, and FEL uses maximum likelihood, whereby the MG94xREV model is used to infer the non-synonymous and synonymous substitution rates for each codon locus after optimizing for branch length and nucleotide substitution parameters. FUBAR uses the Bayesian method to infer the d-non-synonymous replacement rate (dN) and synonymous replacement rate (dS) for each locus based on the given coding sequence arrangement and the corresponding phylogenetic relationship. SLAC assumes that the selection pressure at each locus is constant throughout phylogenesis. SLAC first optimizes branch length and nucleotide substitution parameters under the MG94xREV model, using ML to infer the most likely ancestor sequence at each node of phylogeny (Kosakovsky Pond and Frost, 2005; Murrell et al., 2012; Smith et al., 2015). The significance levels for SLAC, FEL, and MEME were set at 0.1, and the p-value threshold for FUBAR was 0.9. Sites identified by more than two algorithms as positively selected were considered significant and tested for statistical significance using methods described by previous researchers (Li et al., 2020). Positively selected sites were visualized using the Weblogo online tool (https://weblogo.berkeley.edu/logo.cgi).

### 2.6 Statistical analysis

In this study, the sequence information of GETV strains was statistically sorted and analyzed using Microsoft Excel. All bar charts in this study were created using Microsoft Excel.

## 3 Results

### 3.1 GETV exists in several countries in Asia

**Figure 1:**
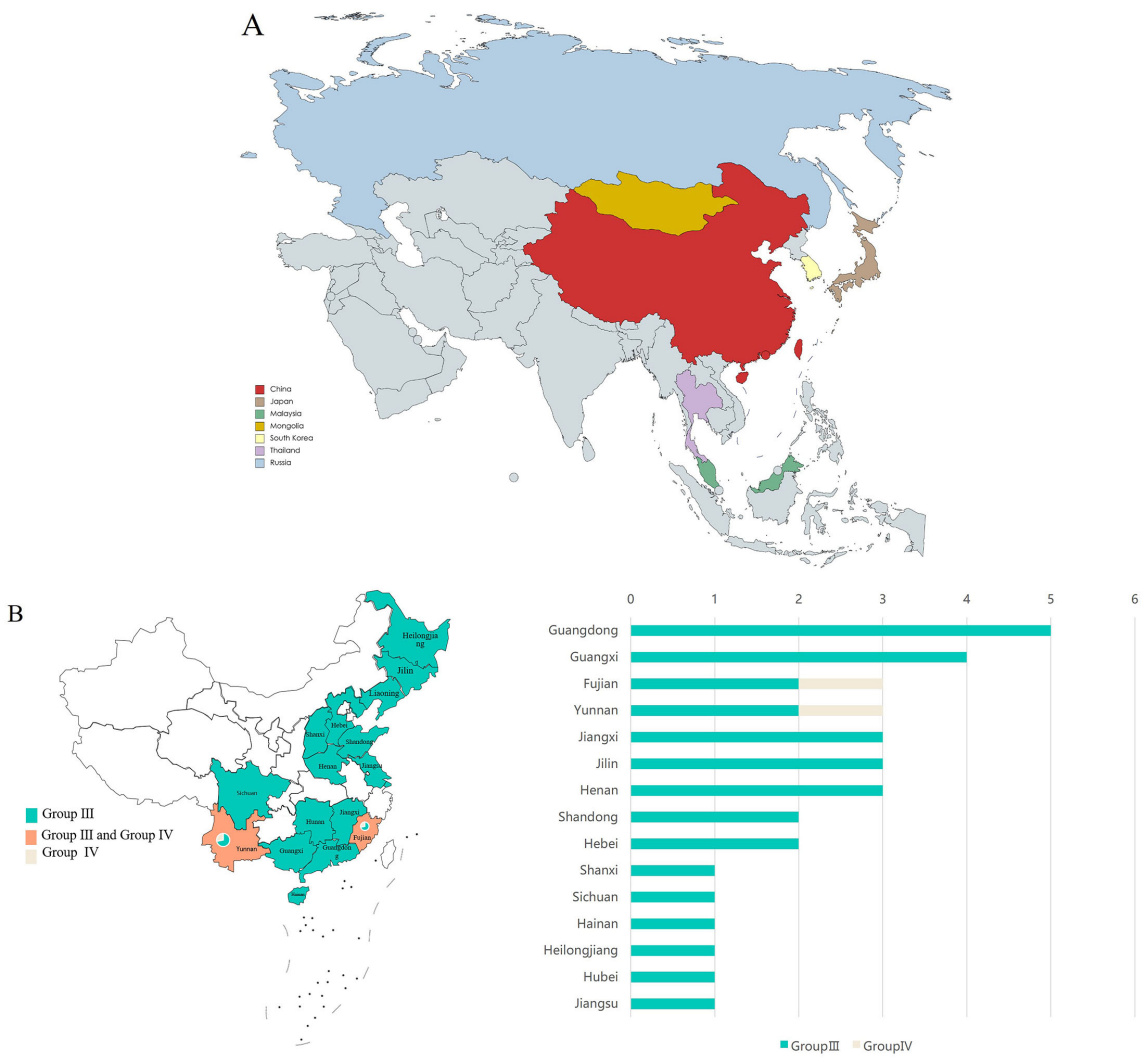
Distribution of GETV. **(A)** Distribution of GETV in Asia. Different color blocks represent different countries. **(B)** Distribution of GETV in China. The left chart shows the distribution of two different genotypes of GETV across 15 provinces in China, with both Fujian and Yunnan provinces containing two genotypes of GETV. The right chart shows the number of sequences uploaded from the 15 provinces. The X-axis represents the number of virus sequences, and the Y-axis represents the name of the province in China.

To investigate the distribution of GETV in Asia, we downloaded all available GETV whole genome sequences from the NCBI database, spanning from 1955 to 2023 (**Supplementary Data 1**). A total of 82 sequences were obtained. According to the sequence information from NCBI, GETV sequences originated from eight countries: China, Japan, Malaysia, Mongolia, South Korea, Thailand, and Russia (**Figure 1A**). The majority of GETV sequences uploaded from China belonged to the GETV Group III strain, mainly from Guangdong,Guangxi, and 13 other provinces (**Figure 1B**). This indicates that the GETV Group III strain is the predominant circulating strain in China, with Guangdong province having the most uploads, likely due to its climate and mosquito species diversity.

**Figure 2:**
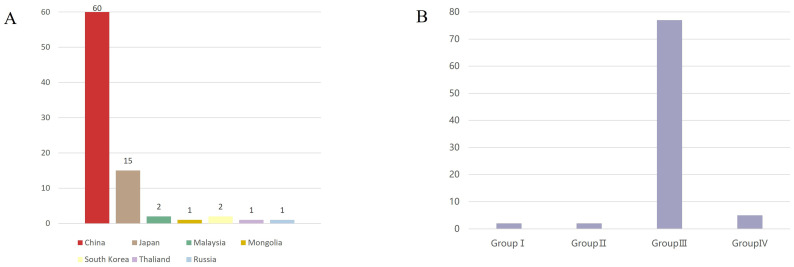
Source and genotypes of GETV. **(A)** Sources of GETV(Asia). The horizontal axis represents the source countries, the vertical axis represents the number of sequences, and different countries are represented by different color blocks. The X-axis represents the country and the Y-axis represents the number of viral sequences **(B)** Genotypes of GETV. The horizontal axis represents the different genotypes of GETV, the vertical axis represents the number of sequences, and Group III is the dominant genotype of GETV. The X-axis represents the four different groupings of GETV, and the Y-axis represents the number of viral sequences.

In all countries where the sequence is uploaded, Among these, the majority of sequences were from China (60 sequences), followed by Japan (15 sequences). Sequences from other countries were fewer: Malaysia (2 sequences), Mongolia (1 sequence), Russia (1 sequence), South Korea (2 sequences), and Thailand (1 sequence) (**Figure 2A**). In terms of genotype, Group III had the highest number of sequences (77), followed by Group IV, and finally Groups I and II, with two sequences each, originating from Malaysia and Japan respectively (**Figure 2B**).

### 3.2 GETV has host diversity

**Figure 3:**
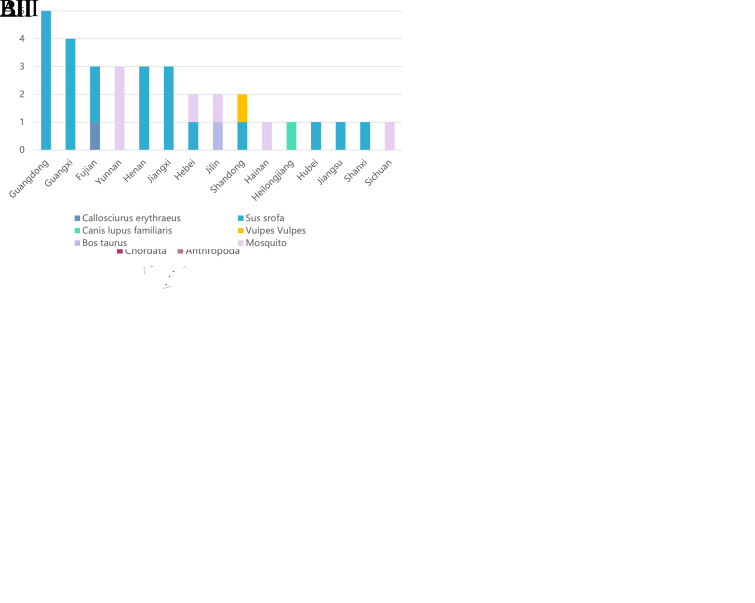
Susceptible host types and distribution. **(A)** Susceptible host types of GETV at the family level (Asia). The X-axis represents the two family levels, and the Y-axis represents the number of viral sequences. **(B)** Distribution of GETV among susceptible hosts in China. Different colors on the map represent different genotypes. **(BI)** The color blocks on the map indicate the host classification, and the circles indicate the specific host species **(BII)** The X-axis represents the number of virus sequences, and the Y-axis represents the provinces of China. **(BIII)** The X-axis represents the provinces of China, and the Y-axis represents the number of virus sequences.

Based on the sequence information of all GETV whole genomes obtained (**Supplementary Data 1**), 81 sequences contained host information, while 5 sequences lacking host information were excluded from the analysis. GETV was found to be transmitted among various hosts through mosquitoes, primarily including vertebrates from the phylum Chordata, subphylum Vertebrata, and the phylum Arthropoda. Vertebrate hosts at the family level included *Bovidae*, *Manidae*, *Equidae*, *Canidae*, *Sciuridae*, and *Suidae*. Arthropoda hosts were exclusively from the *Culicidae* (**Figure 3A**).

In China, GETV-susceptible hosts were mainly distributed across 15 provinces: Guangdong, Guangxi, Fujian, Henan, Jiangxi, Jilin, Yunnan, Hebei, Shandong, Hainan, Heilongjiang, Hubei, Jiangsu, Shanxi, and Sichuan (**Figures 3BI-II**). Provinces with infections in both vertebrates and arthropods included Hebei and Jilin. Provinces with infections only in vertebrates included Guangdong, Guangxi, Fujian, Henan, Jiangxi, Shandong, Heilongjiang, Hubei, Jiangsu, and Shanxi. Provinces with mosquito-only infections were Hainan and Sichuan (**Figure 3BIII**).

**Figure 4:**
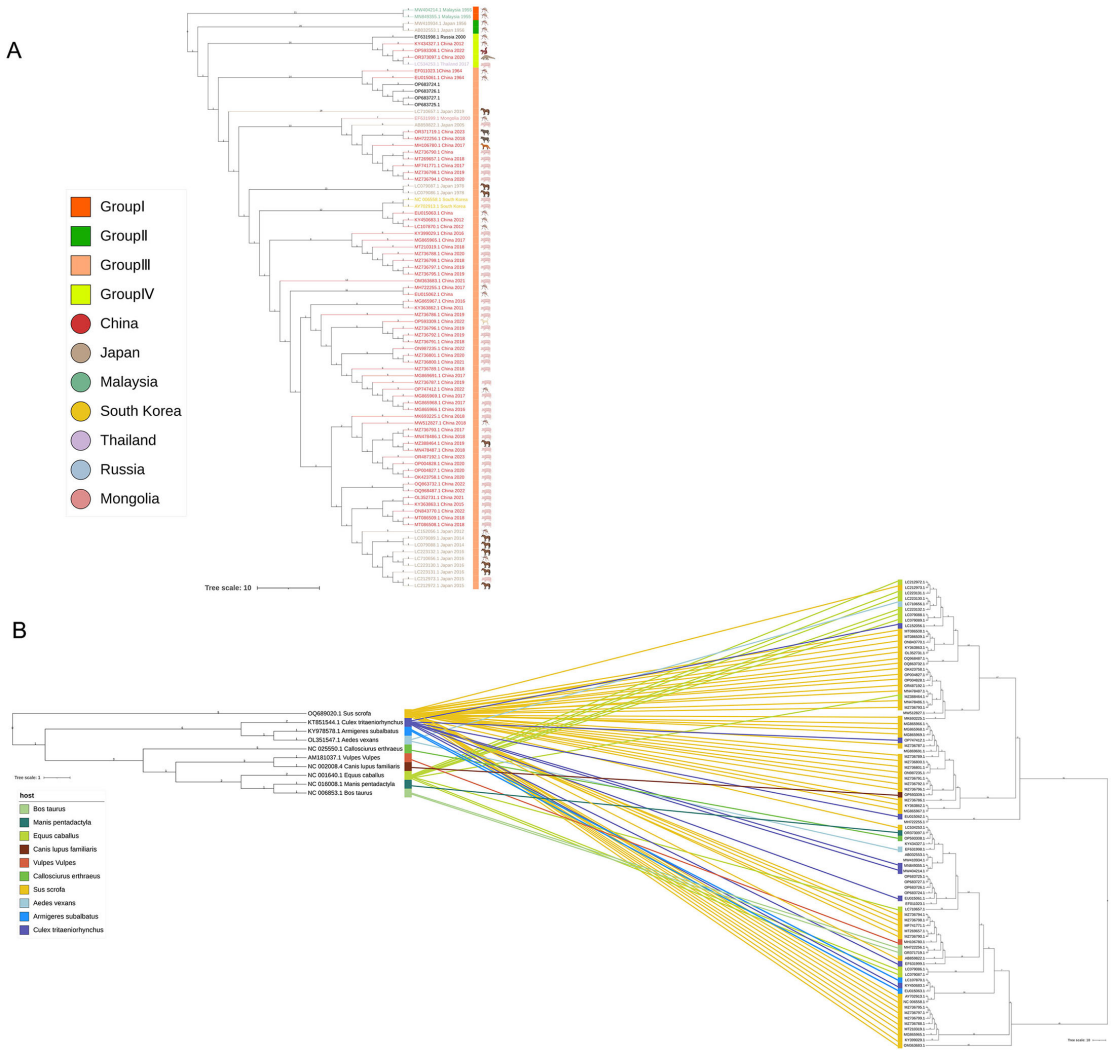
Complete genome phylogenetic analysis and entanglement relationship between GETV and hosts. **(A)** The squares represent the groupings, and the circles represent the country of origin. **(B)** A phylogenetic tree was constructed using 82 full-genome sequences of GETV, with the best model selection being GTR+G+I. Additionally, 10 different host mitochondrial DNA sequences were used for tree construction, with the best model selection being TPM2u+F+R3. Branches and associations are colored according to host types. Tree scale 1 represents the unit in which each unit length corresponds to 1 nucleotide or amino acid substitution; Tree Scale 10 represents the unit in which each unit length corresponds to 10 nucleotide or amino acid substitutions.

Downloading the mitochondrial cytb gene sequences of GETV hosts from the NCBI database, and based on the sequence information of GETV and hosts from NCBI, phylogenetic trees were constructed for the 82 GETV whole-genome sequences (**Figure 4B**, right) and the 10 hosts whole-genome sequences (**Figure 4A**, left). The entanglement relationships between hosts and viruses were also constructed (**Figure 4B**). By performing multiple sequence alignments and phylogenetic analyses, a total of 82 whole-genome sequences from NCBI were studied. Phylogenetic analysis shows that GETV has gradually evolved into four distinct genetic groups, of which the group I originated from the original GETV strain of mosquitoes found in Malaysia (MM2021), which is located at the root of the phylogenetic tree, indicating that it is the oldest strain. The group II included only two strains isolated from Japanese mosquitoes in 1956. The group IV included one strain isolated in 2000 from mosquitoes in Russia, one strain isolated in 2017 from wild boar in Thailand, and three strains isolated from China. Of the three sequences from China, one strain was isolated from mosquitoes in Yunnan in 2012, one from pangolins in 2020, and one from red-backed squirrels in Fujian Province in 2022. The remaining sequences all clustered into Group III in the phylogenetic evolutionary tree (**Figure 4A**). Notably, there were several intertwined linkages between mosquitoes, horses, and pigs in infection with GETV, which may be the result of frequent co-infections in these species.

### 3.3 Transmission and adaptive evolution of GETV

**Figure 5:**
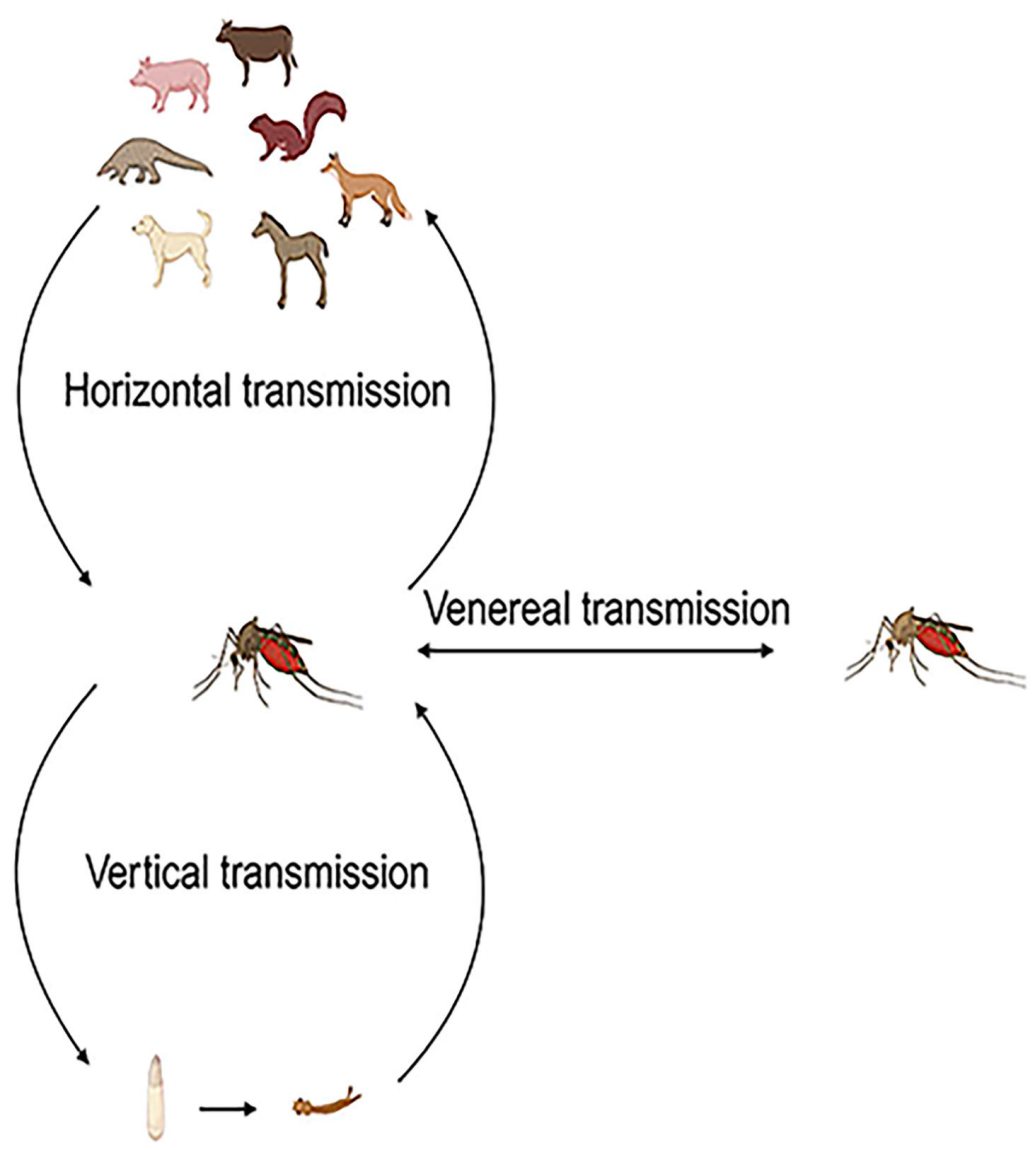
Vector transmission of GETV. The vector transmission of GETV occurs through three main modes: horizontal transmission between vertebrate hosts and vectors, vertical transmission from infected female mosquitoes to their offspring, and sexual transmission from infected female mosquitoes to male mosquitoes and vice versa.

Based on the information uploaded to NCBI, it is evident that mosquitoes can horizontally transmit GETV to cows, pangolins, horses, dogs, squirrels, and pigs. The transmission of GETV is mainly caused by mosquito bites and thus spread to various hosts. Vertical transmission of arboviruses in mosquitoes (from infected females to their offspring) is considered a mechanism for maintaining the virus during unfavorable horizontal transmission conditions among vertebrate hosts, such as harsh winters, dry conditions, and interepidemic periods limited by herd immunity, and may influence the epidemiology of arbovirus infections (**Figure 5**) (Lequime et al., 2016).

**Figure 6:**
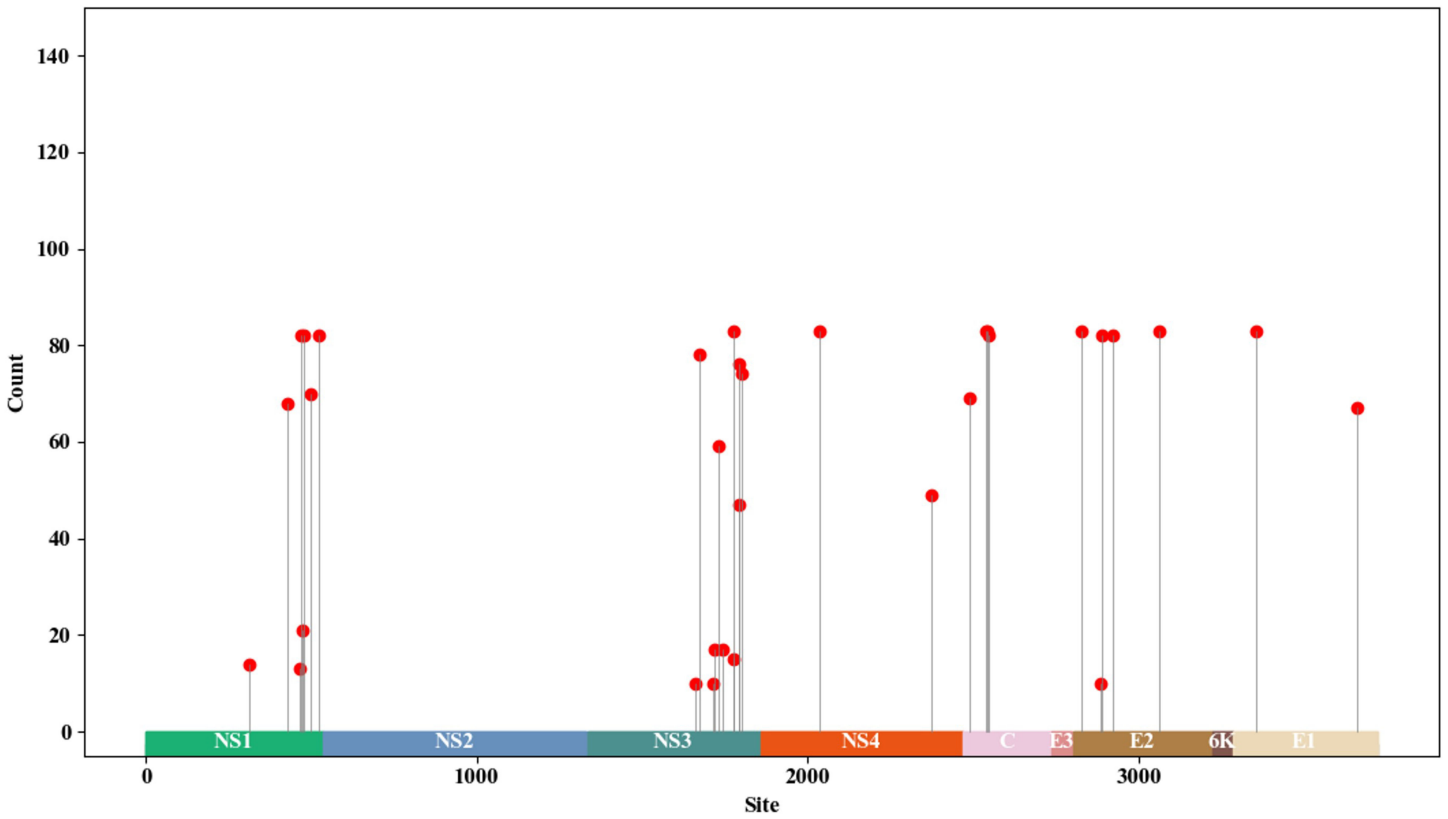
Evolution of amino acid adaptive sites in GETV. Single nucleotide variants in each gene of GETV strains obtained from NCBI. Amino acid variation in polyproteins is distributed along the genome. The 82 GETV complete genome sequences downloaded from the NCBI database show only mutations that appear in more than 10 serial lines. NSP1–4 represents non-structural proteins, C, E3, E2, 6K, E1 represent structural proteins, and UTR represents untranslated regions.

**Figure 7:**
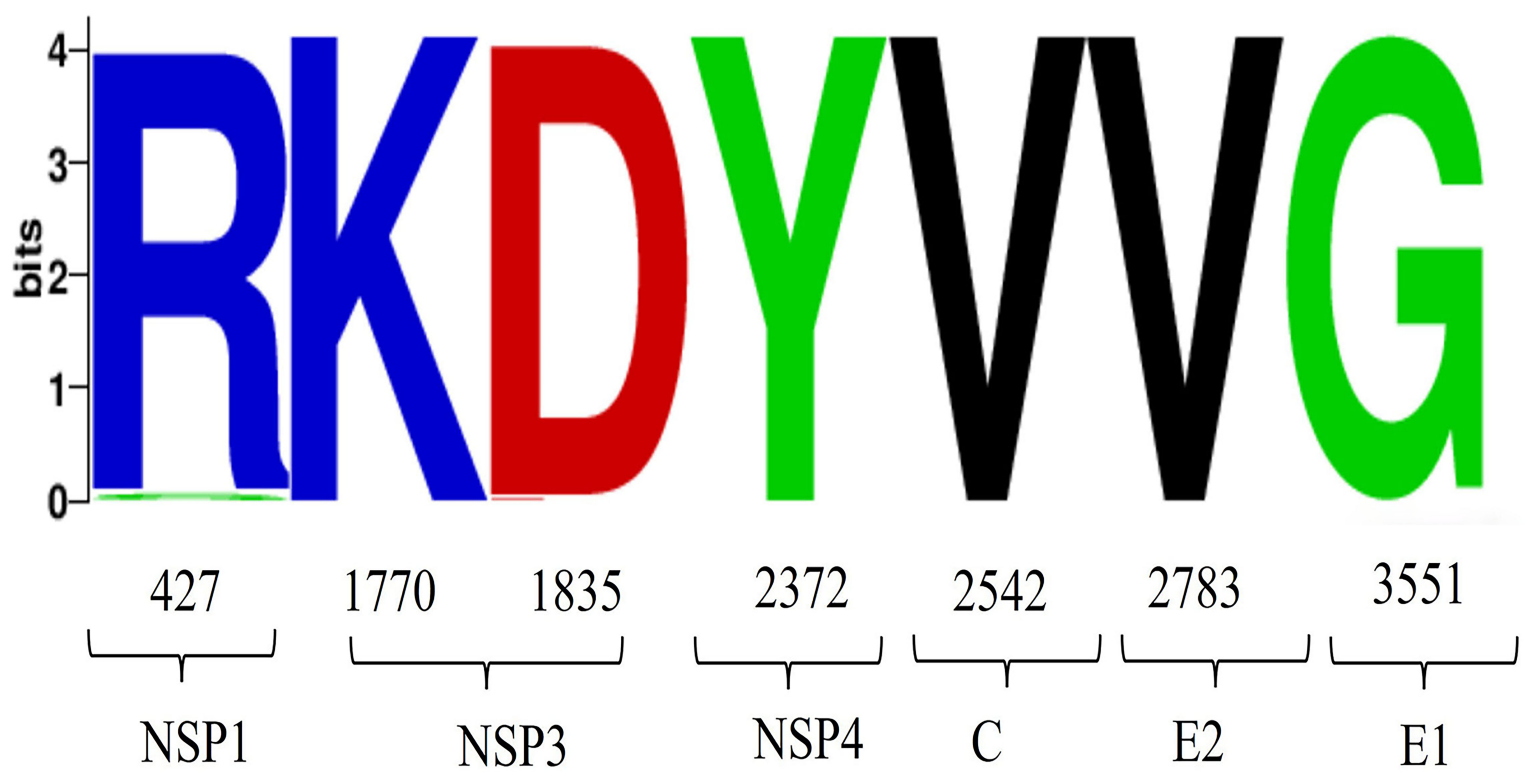
Analysis of amino acid variations in GETV. Visualization analysis of the amino acid positive selection sites detected in **Table 1**, with the horizontal axis representing the positions of amino acids.

We analyzed the mutation sites in the 82 GETV whole genome sequences, revealing that most mutations occurred in non-structural proteins, with eight mutations in the NSP1 gene and 11 in the NSP3 gene. Mutations were also detected in structural proteins, although fewer than in non-structural proteins, with four mutations in the C gene and five in the E2 gene (**Figure 6**). Under positive selection, we identified seven positively selected sites in the 82 GETV whole genome sequences. The positions of these sites in the whole genome sequence were: site 427, site 1770, site 1835, site 2372, site 2542, site 2783, and site 3551 (**Table 1**). Visualization of amino acid variations encoding adaptive sites showed that only at site 427, there was an amino acid variation where arginine changed to serine (**Figure 7**).

**Table 1:** Analysis of positive selection sites in GETV amino acids.

Protein	Site	MEME (p-value)	FEL (p-value)	FUBAR (Post.Pro)
NSP1	427	0	0.002	1
NSP2	N/A	N/A	N/A	N/A
NSP3	1770	0.06	0.045	0.902
	1835	0.01	0.1	0.935
NSP4	2372	0.07	0.05	0.98
C	2542	0.1	0.078	0.99
E3	N/A	N/A	N/A	N/A
E2	2783	0	0.02	0.996
6K	N/A	N/A	N/A	N/A
E1	3551	0.07	0.097	0.975

Recombination analysis was performed on the coding region sequences, excluding recombinant sequences and sequences containing degenerate and ambiguous bases. A total of 82 sequences were included in the positive selection site analysis. Positions identified by at least two out of three algorithms were considered valid, resulting in the detection of seven positive selection sites.

N/A, no available.

## 4 Discussion

Due to the ability of *Alphaviruses* to cross species barriers multiple times, the transmission of viruses within the *Alphavirus* genus poses a significant risk to both animal and human health. Bannai et al. demonstrated that GETV can be transmitted from horses to pigs (Bannai et al., 2017). Therefore, we examined the characteristics of GETV in terms of epidemiology, host diversity, and adaptive evolution.

In our study, by integrating sequence information uploaded to NCBI, we found that the GETV full-genome sequences mainly come from eight countries: China, Japan, Malaysia, Mongolia, South Korea, Thailand, Russia, and others. The sequences from Russia are geographically located in the northeastern part of Asia. Globally, since the original GETV strain (MM2021) was first isolated from Malaysia in 1955, GETV has been isolated in 13 countries, including Indochina countries in Asia (Vietnam, Thailand, Cambodia), Southeast Asian countries (Malaysia, Philippines), South Asian subcontinent (India, Sri Lanka), Australia, Japan, South Korea, Mongolia, China, and Russia (Ksiazek et al., 1981; Peiris et al., 1994; Bryant et al., 2005; Liu et al., 2017; Li et al., 2021). China is the country with the most uploaded GETV sequences, followed by Japan. China, Japan, and South Korea serve as the hubs for GETV transmission, covering the vast majority of strains. These three countries have rapidly developed and extensive livestock industries, with most animal products sold to neighboring regions, thereby accelerating the local spread of GETV. Studies on the transmission pathways of GETV have shown that Malaysia, Japan, and Yunnan province in China are the three main transmission sites of the virus. GETV probably spread from Malaysia to Japan in 1917, from Japan to Hainan province in China in the 1960s, from Japan to Yunnan province in China in the 1970s, and then through Yunnan province in China to Hebei province, Shanghai, Gansu province, and Sichuan province, China (Bryant et al., 2005; Li et al., 2017; Shi et al., 2022). In these countries, numerous convergence points in the GETV transmission routes form a closed co-circulation loop (Shi et al., 2022). This reminds us that at the moment of increasingly globalization, various countries have close exchanges, and the conditions for the dissemination of GETV should be strictly controlled by the GETV detection methods for the import and export of animal products of various countries.

In our study, through multiple sequence alignments and phylogenetic analysis of known GETV full genomes, the results show that GETV can be divided into four genotypes (Group I-IV). Group I consists of the original GETV strain (MM2021) isolated in Malaysia in 1955. Group II includes only the strain isolated from Japanese mosquitoes in 1956. Group IV comprises one strain isolated from mosquitoes in Russia (LEIV-16275-MAG, 2000), one strain from Yunnan province, China (YN12031, 2012), one strain isolated from pangolins in China (2020), one strain from red-backed squirrels in Fujian, China (Rbsq202206, 2022), and one strain from Thailand (SW). This indicates that Group IV has still spread in China in recent years and has been found in new hosts. The continued spread of Group IV virus indicates that GETV is breaking through the traditional host boundaries. This highlights the need for preventive measures against GETV in animals to prevent outbreaks. The remaining strains are classified into Group III. In terms of numbers, Group III is the dominant strain of GETV. The absolute advantage of the number of Group III strains reveals the adaptive evolution of GETV during transmission to promote the continuous transmission of Group III strains. The phylogenetic tree results show that Groups I, II, and IV have become stable viral populations in local regions, while the geographical range of Group III has rapidly expanded. Therefore, enhancing the detection and monitoring of GETV infections is crucial for the timely identification of animal outbreaks, thereby reducing economic losses in livestock. Additionally, although GETV outbreaks causing human diseases have not been reported, monitoring people involved in livestock farming (especially those working with horses and pigs) in GETV-endemic areas is particularly important to avoid cross-species transmission to humans. In China, a total of 15 provinces have uploaded full-genome sequences of GETV. The first isolation of GETV in China was from a Culex mosquito collected on Hainan Island in 1964, later identified as GETV. Guangdong and Guangxi provinces have uploaded the most sequences, likely due to the extensive livestock industry and widespread GETV infection in pigs in these regions. In our study, two provinces uploaded sequences from two different GETV genotypes, one of which is Yunnan province. Among the provinces where GETV has been found, Yunnan has been conducting GETV-related research earlier and has isolated the most strains. Yunnan is located in tropical and subtropical regions, with year-round high temperatures suitable for mosquito survival, providing favorable conditions for the circulation of GETV. GETV strains have been isolated in multiple locations in Yunnan, such as Tengchong and Ruili (Zhang et al., 2020).

It has long been widely believed that GETV primarily infects horses and pigs (Bannai et al., 2019; Sun et al., 2022). Pigs are considered the primary amplifying hosts because they develop viremia after being infected with GETV (Kuwata et al., 2018). Horses are important hosts for the amplification and circulation of GETV (Bannai et al., 2015). In our study, the host species of GETV include ten different types: mosquitoes (*Culex*, *Aedes*, and *Armigeres*), cattle, pangolins, horses, dogs, foxes, squirrels, and pigs. Notably, from 2014 to 2023, GETV whole genome sequences have been isolated from various hosts each year. Specifically, in 2020, a strain containing the complete genome sequence of GETV was isolated from a pangolin, and in 2022, strains containing the whole genome sequences of GETV were isolated from a squirrel and a dog. The continual discovery and isolation of GETV strains in new host species indicate that the range of hosts for GETV is expanding, and the virus is spreading among different hosts. In our study, by intertwining the phylogenetic tree of host species with the phylogenetic tree of GETV whole genome sequences, we further confirmed the co-circulation of GETV between mosquitoes and other animals. The infection in different species is essential for maintaining the zoonotic transmission cycle of GETV in nature. In recent years, new species infected with GETV have been frequently reported in China. However, due to the lack of in-depth research on the cross-species transmission events of GETV, GETV has only been around for 70 years, so the scale of evolutionary events of host and virus differentiation varies by orders of magnitude. In conclusion, our study suggests that cross-species transmission of GETV in recent years should be taken seriously to prevent GETV outbreaks in new hosts.

Azerigyik et al. (2023) have shown that mosquitoes are the primary vectors of GETV. Mosquitoes carrying GETV have been isolated from four genera and eight species, including four species of Culex (*Culex tritaeniorhynchus*, *Culex pseudovishnui*, *Culex gelidus*, and *Culex annulirostris*), one species of Aedes (*Aedes albopictus*), one species of Anopheles (*Anopheles sinensis*), one species of Armigeres (*Armigeres subalbatus*), and one species of mixed mosquitoes. Among these, *Culex tritaeniorhynchus* and *Armigeres subalbatus* are widely distributed in China, and often found around rural livestock pens and breeding farms. They have high reproductive capacity and large populations, making them considered dominant mosquito species with significant importance in carrying and transmitting GETV. Hubálek et al. (2014) have also isolated GETV from biting midges *Culicoides* spp. These studies indicate that the variety of mosquito species transmitting GETV is continually expanding, and the diversity of vectors has increased. Surveys conducted among febrile patients and healthy individuals have found the presence of GETV-specific IgM and IgG antibodies in some humans. The antibody titers in febrile patients were significantly higher than in healthy individuals, suggesting that GETV infection may be associated with human diseases (Li et al., 2019; Lu et al., 2019). GETV-specific antibodies have also been detected in serum samples from chickens, ducks, goats, and birds. These studies collectively indicate that the types of hosts infected by GETV are increasing, and the host range is expanding. Seroepidemiological tests in animals or humans infected with GETV can be detected by serological methods, such as neutralization test (NT) (Matsumura et al., 1981), complement fixation test (CFT) (Li et al., 2016), hemagglutination inhibition test (HIT) (Hohdatsu et al., 1990), enzyme-linked immunosorbent assay (ELSA) (Kuwata et al., 2018), etc. ELSA is by far the most commonly used method. At present, there is no commercially available antigen detection method, so the etiological detection of GETV mainly includes viral gene amplification and virus isolation, viral gene amplification mainly includes reverse transcription-polymerase chain reaction (RT-PCR) (Wang et al., 2018), and for virus isolation, the blood-sucking insect specimens or animal specimens that can be collected are ground and centrifuged, and the supernatant is inoculated into tissue culture cells (Simpson et al., 1975).

Studies have shown (Gorchakov et al., 2012) that single nucleotide substitutions can significantly alter *Alphavirus* vector specificity and pathogenicity. We conducted whole-genome mutation, selection, and adaptive evolution analyses of GETV, identifying 21 amino acid mutations in non-structural proteins and 11 in structural proteins. The non-structural protein mutations include eight in NSP1 (M313V, I427T, Q465R, E468K, M475T, S478G, A498V, T521A), 11 in NSP3 (P1661S, A1672T, V1713T, Q1717R, I1730T, A1742T, Q1774P, L1775S, I1790T, T1793P, G1799E), and two in NSP4 (R2036Q, T2372A). The structural protein mutations include four in the capsid (F2487Y, S2537P, T2544A, T2546A), five in E2 (S2826F, H2885Y, T2889V, I2921T, D3061N), and two in E1 (K3353M, T3659M). Since the interaction between E1 and E2 is crucial for viral budding and entry, studying the amino acid variations in E1 and E2 is essential (Mukhopadhyay et al., 2006). At the same time, this study revealed the molecular adaptation mechanism of GETV through positive selection site analysis, and identified seven forward selection sites. It is worth noting that E2 and E1 proteins are labeled as potential selection pressure sites by multiple algorithms. These mutations provide evidence of genetic diversity in the GETV genome. Further research into the adaptive evolution of GETV amino acids through positive selection analysis identified seven positively selected sites, encoding Arg, Lys, Asp, Tyr, Val, Val, Gly. Hydrophilic amino acids (e.g., Arg, Lys, Asp) typically interact with the aqueous environment on protein surfaces, while hydrophobic amino acids (e.g., Val) are more likely to be found inside proteins, helping to form a stable hydrophobic core.

Our study offers novel insights into the distribution of GETV in Asia, the diversity of susceptible hosts, and adaptive sites. As GETV continues to evolve and its host range expands, comprehensive monitoring of GETV is necessary to prevent outbreaks from escalating into pandemics, especially in countries like China, Japan, and South Korea, where GETV is widespread.

In this study, we found that China is the country with the most uploaded sequences in the world, although we know that the number of GETV sequences is not enough compared to that of other alphaviruses, which may also be due to the delay in sequence upload in some countries and regions. In the future, when the number of GETV sequences increases, the study of GETV may be more complete to better prevent the epidemic of GETV. It is undeniable that this study also has certain limitations, due to the lack of research on GETV, the coverage of the whole genome sequence of GETV obtained from the NCBI database is limited, and secondly, the study only integrates genomic and geographical distribution data, but does not combine serological investigation, host population distribution, disease incidence, etc., which has certain limitations, despite the above challenges and limitations, this study systematically reveals the three key characteristics of GETV in China, and provides an important scientific basis for the formulation of prevention and control strategies. First, the continuous selection pressure of key sites such as NSP1 protein suggests that the virus may be breaking through the host immune barrier. Second, the risk of zoonoses has escalated, and antibodies have been detected in healthy people, indicating that potential public health threats are accumulating. Finally, it should be noted that group III virus has become a regional epidemic in China, which suggests that we need timely prevention of GETV. In summary, the number of vertebrate infections in GETV in China has increased rapidly in recent years, and the host range has been expanding. However, the threat posed by the Chinese GETV has not yet attracted sufficient attention. In order to prevent and control GETV, a series of relevant measures must be taken.

## 5 Conclusion

In this study, we identified that GETV is rapidly expanding its host range and geographic distribution at a high evolutionary rate. Coevolutionary analysis of GETV and its host showed that GETV had cross-species transmission events. Finally, we identified important adaptive and positive selection sites. Our study shows that GETV is widely distributed in China and is constantly infecting new hosts.

## Data Availability

The original contributions presented in the study are included in the article/**Supplementary Material**. Further inquiries can be directed to the corresponding authors.
